# Antimicrobial Use and Resistance in Surplus Dairy Calf Production Systems

**DOI:** 10.3390/microorganisms10081652

**Published:** 2022-08-16

**Authors:** Poonam G. Vinayamohan, Samantha R. Locke, Rafael Portillo-Gonzalez, David L. Renaud, Gregory G. Habing

**Affiliations:** 1 Department of Veterinary Preventive Medicine, The Ohio State University, Columbus, OH 43210, USA; 2 Department of Population Medicine, University of Guelph, Guelph, ON N1G 2W1, Canada

**Keywords:** antimicrobial use, antimicrobial resistance, surplus calves, antimicrobials, veal, bovine respiratory pathogens, commensal bacteria, foodborne pathogens

## Abstract

Surplus calves, which consist predominately of male calves born on dairy farms, are an underrecognized source of antimicrobial-resistant (AMR) pathogens. Current production systems for surplus calves have important risk factors for the dissemination of pathogens, including the high degree of commingling during auction and transportation and sometimes inadequate care early in life. These circumstances contribute to an increased risk of respiratory and other infectious diseases, resulting in higher antimicrobial use (AMU) and the development of AMR. Several studies have shown that surplus calves harbor AMR genes and pathogens that are resistant to critically important antimicrobials. This is a potential concern as the resistant pathogens and genes can be shared between animal, human and environmental microbiomes. Although knowledge of AMU and AMR has grown substantially in dairy and beef cattle systems, comparable studies in surplus calves have been mostly neglected in North America. Therefore, the overall goal of this narrative review is to summarize the existing literature regarding AMU and AMR in surplus dairy calf production, highlight the management practices contributing to the increased AMU and the resulting AMR, and discuss potential strategies and barriers for improved antimicrobial stewardship in surplus calf production systems.

## 1. Introduction

The rise in antimicrobial resistance (AMR) is a major public health crisis [1], and food animals are important reservoirs of AMR bacteria [2]. However, most studies on AMR in food animals focused on either poultry, swine, dairy, or feedlots production systems, with scarce attention paid to surplus calf production. Interestingly, Salaheen et al. [3] found that about 70% of veal calves had resistomes containing AMR genes conferring resistance to multiple medically important antimicrobials. It is therefore important to study the contribution of surplus dairy calves to overall AMR spread.

**Figure 1:**
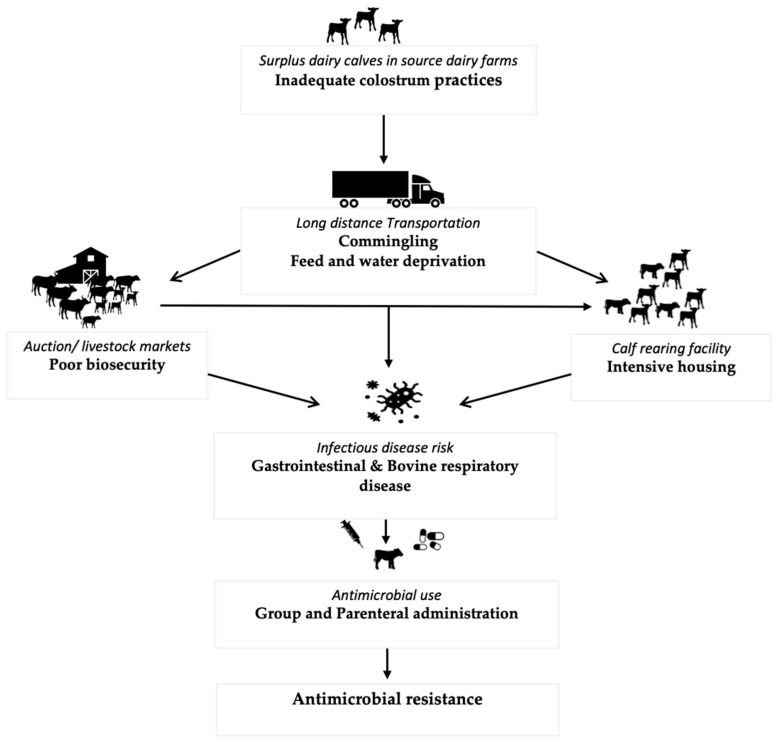
Events and causal pathways in surplus calf production leading to increased infection risk and subsequent AMU.

Surplus dairy calves are calves born on dairy farms that are either unsuitable or not required to replace the milking herd [4]. Most of these calves are male and are either sold for “bob” veal (marketed at <3 weeks of age), veal (milk-fed or formula-fed veal raised mainly only on a milk-based diet with some amount of grain and marketed at 20 weeks of age; grain-fed veal raised mainly on a milk-based diet until 6–8 weeks of age before transitioning to a grain-based diet and marketed at 8 months of age), or dairy beef (marketed at 12–14 months of age). Recently, however, there has been an exponential increase in the use of beef semen in Holstein cows leading to an increase in the number of surplus cross-bred calves [5]. The production stages in surplus calves involve sometimes long-distance transportation to the auction or livestock markets from where the calves are either sent for slaughter or are purchased by the calf raisers to be reared for a certain amount of time depending on the production system type. Throughout these stages, surplus calves experience many health challenges [6,7,8,9,10,11,12,13], leading to an increased risk of disease and subsequent antimicrobial use (AMU) as represented in Figure 1 [4].

AMU in the surplus calf production operations represents an unquantified risk to human health through the foodborne transmission or environmental dissemination of AMR bacteria and the genetic elements mediating resistance. The academic literature has devoted relatively little effort to characterizing the types, indications, and quantity of antimicrobials used and the emerging AMR within surplus calf production systems. In North America, research has characterized AMU among dairy cattle [14,15,16], but few studies focused on AMU in dairy calves [17]. Only a single manuscript in North America has detailed AMU in surplus calf production systems [18]. By comparison, a plethora of research in Europe [19,20,21,22,23] has characterized or identified AMU in surplus calf industries. An overview of the current state of AMU and AMR among commensal and pathogenic bacteria in surplus calves is important to develop scientifically supported and applicable measures to curb AMU and reduce the risk of AMR. Therefore, the overall goal of this review is to (a) summarize the current situation of AMU and AMR in surplus dairy calf production systems, (b) highlight the management practices contributing to the increased AMU and the resulting AMR, and (c) discuss potential strategies and barriers for improved antimicrobial stewardship in surplus calf production systems.

## 2. Individual and Group Antimicrobial Use in Surplus Calf Production

Individual therapies, primarily consisting of parenteral administration, are mostly used for the treatment of respiratory and gastrointestinal diseases [19], and the majority of AMU happens in the first two months of life [18]. Within the peer-reviewed literature, the reported frequencies of individual AMU in surplus calf systems are typically higher than in other types of animal production. For instance, 61% to 87% of calves received at least one injection of an antimicrobial in their production period [19,24,25]. The level of individual AMU is likely a reflection of the disease incidence. The higher burden of disease has been confirmed through objective health scoring—roughly 85% of calves at a veal facility had at least one day with an abnormal fecal consistency score in the first 28 days after arrival [26]. In addition, others have found a similar disease burden with calves having an abnormal respiratory score for on average 7% of the days the calves were observed at a veal facility [27]. Although prior studies confirm a uniquely high disease burden, AMU could likely be reduced through more targeted AMU. For instance, roughly 40% of replacement heifer producers reported typically using antimicrobials in cases without systemic signs of disease [28]. In another study, Uyama et al. [29] reported that about 96% and 74% of the Canadian dairy producers used antimicrobials to treat respiratory and diarrhea diseases, respectively, in pre-weaned dairy calves. However, less than half of the producers had any written treatment protocol for calf diseases [29].

In addition to individual administration, producers often use group administration of antimicrobials as a prophylactic measure for calf health on arrival due to high disease risks in the first weeks of life. In most contexts, the primary indications for group antimicrobial therapy are (1) metaphylaxis of disease for arriving groups of calves, (2) treatment of gastrointestinal disease and (3) treatment of respiratory disease. In the U.S., group administration of antimicrobials in the feed or water requires a Veterinary Feed Directive or prescription, respectively. Calf producers often use group administration of antimicrobials for the treatment, control, or prevention of disease. In the U.S., 77% of surveyed veal producers reported that they use blanket therapy for the treatment of either diarrhea or pneumonia [30], and group antimicrobial administration constituted 24.1% of the doses of antimicrobials on Ohio veal farms [18]. In Europe, the frequency and/or quantity of group administration of antimicrobials varies widely; investigators reported that 13.4% of farms in Switzerland used group treatments [31], while in France and Belgium, 98% of antimicrobial treatments and antimicrobial doses were group treatments rather than individual treatments [19,32]. By contrast, 85% of antimicrobial doses were administered parenterally rather than in a blanket group therapy on farms in Denmark, potentially reflecting official Danish guidelines that caution against oral administration [23].

### 2.1. Common Antimicrobials Used in Surplus Calf Production

Beyond the quantity of antimicrobials, the antimicrobial selection is an important consideration for optimal antimicrobial stewardship. The World Health Organization has categorized antimicrobials as either critically important antimicrobials (CIA), highly important (HPA), or important (IA) according to the importance for human medicine. In addition, 3rd generation cephalosporins, fluoroquinolones, and macrolides are categorized as highest-priority (HP-CIAs) [33]. In the U.S., fluoroquinolones (e.g., enrofloxacin) are available to treat respiratory disease in all classes of beef cattle and dairy heifers under 20 months of age but are prohibited for use in veal calves. Third-generation cephalosporins are labeled for bovine respiratory disease and are used on dairy-beef and veal operations for treatment. Veal producers in the U.S. used a mean of 1.0 defined doses of ceftiofur per 100 calves per day, which comprised 3.3% of the individual treatments, and fluoroquinolones were reportedly not used [18]. In European countries, fluoroquinolones and cephalosporins represented 1.9% and 0.1% of the total doses of AMU, respectively [20]. Among the three classes of HP-CIAs (fluoroquinolones, 3rd-generation cephalosporins, and macrolides), macrolides appear to be the most frequently used. Specifically, in a study conducted in Belgium, oral and long-acting parenteral macrolides comprised 11% and 28% of total use, respectively, in part owing to the respiratory disease treatment label and reported effectiveness against *Mycoplasma bovis*, a common cause of respiratory disease in veal calves [20]. In addition, macrolides were commonly used as individual treatments (4.7% of individual treatments) [18], and macrolides were among the three most used antimicrobials in veal calves in France [22]. Although macrolide use may become a target for improved stewardship, increased use was a protective factor against mortality, and the use of macrolides are likely important for reducing the negative impact of respiratory disease [34].

The largest number of doses of antimicrobials used on veal farms were those that were more commonly administered in a group fashion. Antimicrobials most used for group therapy and administered in the water included chlortetracycline, neomycin, amoxicillin, and sulfmethoxazole/trimethoprim [18]. Of course, the types of antimicrobials used for group treatments in different countries in part reflect differing regulatory availability of antimicrobials. For instance, doxycycline was the second most used antimicrobial in Belgium [20], and colistin was the second most used antimicrobial in veal herds in the Netherlands [35].

### 2.2. Quantification of Antimicrobial Use in Surplus Dairy Calves

Quantification of AMU on farms is necessary to measure and monitor the effect of AMU reduction strategies. Quantified estimates of AMU are frequently expressed as a treatment incidence rate, i.e., the number of doses per units of animal-time (e.g., 1000 calf-days). Calculation of the rate requires a definition of a “dose” of antimicrobials (e.g., defined daily doses) and assumed standard animal weight. Methods and assumptions for the calculation have not been standardized in the U.S., but dose definitions have been published in Canada [36] and Europe [37]. Comparisons of AMU estimates between published manuscripts are inherently difficult and particularly difficult for growing calves. For instance, Cheng et al. [18] used an estimate of weight based on carcass weights at slaughter and assumed growth rates, whereas other reports use, for instance, a standard weight of 160 kg [34] or 60 kg [38], likely underestimating the number of doses for lighter calves (i.e., neonates) when AMU is most common. Nonetheless, available research suggests that dosing rates are substantially higher among surplus calf production systems. Among Ohio veal calf herds, veal calves received a mean of 35 doses per 100 calves per day, whereas dosing rates ranged between 1.44 and 2.08 per 100 days on beef or dairy production systems [15,39,40]. Similarly, the animal defined daily dose (ADD) in Belgium veal herds was almost double that of the ADD of dairy and beef herds for parental antimicrobials [41]. The quantity of AMU can substantially vary between farms. For instance, Bokma et al. [34] showed that the dosing rate was approximately 60% higher on farms managed by one veal company relative to a different company. Across 78 farms, the doses used per calf-year ranged from 10 to over 50 [20], and the number of treatments per calf ranged from 2.8 to 15.9 on farms in France [22]. Similarly, the treatment incidence among Ohio, USA veal farms ranged from 20.4 to 54.4 doses per 100 calf-days. The significant between-farm variation suggests there is an opportunity for a substantial reduction in the dosing rate of antimicrobials

### 2.3. Major Risk Factors Associated with Antimicrobial Use in Surplus Calf Production

Research evaluating the risk factors for AMU in surplus calves has mostly focused on the European and Canadian veal industries. As shown in Table 1, there are multiple risk factors associated with AMU in surplus calves. Clearly, the quality of the arriving calves and the management practices in the calf rearing facility plays a critical role in the occurrence of disease and the use of antimicrobials. Therefore, interventions to reduce the incidence of disease and AMU will require collaborations across multiple segments of the production chain, including dairy farms, transporters, and growers.

## 3. Surplus Calf Production System as Reservoirs of Major Antimicrobial Resistant Pathogens

The selective pressure exerted by antimicrobials is one of the main drivers of AMR in commensals and zoonotic enteropathogens [49,50,51,52]. AMR carriage in surplus calves is a public health concern due to the potential for transmission to humans via direct contact, environmental contamination, or contamination of food. In 2016, the Wisconsin Department of Health and the US Centers for Disease Control and Prevention identified dairy calves sold at livestock markets as the source of the multidrug-resistant *Salmonella* Heidelberg outbreak in humans [53]. AMR is also a concern for animal health as resistance in animal pathogens reduces the effectiveness of drugs used in veterinary medicine. Direct contact with surplus calves or their environments could transmit AMR to other animals (e.g., via livestock trailers or auctions). Several studies report that among all cattle sectors, the highest levels of AMR in commensals and pathogens are found in veal production systems [54,55,56]. Specifically, bovine respiratory pathogens, enteric pathogens, and foodborne infectious agents are the major group of organisms that have significant health and economic consequences in animals and humans and are widely studied for the presence of AMR in surplus calf production systems. The following paragraphs address each of these categories directly.

### 3.1. Bovine Respiratory Pathogens Play a Key Role in Overall Antimicrobial Use and Antimicrobial Resistance

Bovine respiratory disease (BRD) has been recorded as the leading cause of morbidity and mortality in pre-weaned calves in the United States [57], wherein in an analysis that involved 43,739 calves, 10.5% were diagnosed with BRD [57]. Bovine respiratory disease (BRD) is a multifactorial disease complex involving numerous bacterial and viral agents which act in synergy with stressors such as nutrition, weaning, transportation, and rearing environment. Members of the *Pasteurellaceae* family (*Mannheimia haemolytica, Pasteurella multocida, Histophilus somni,* and *Mycoplasma bovis*) are often described as secondary infectious agents but are also considered as commensal organisms in the upper respiratory tract [58]. Owing to the high infection pressure after transportation, pro- and metaphylactic treatments are frequently used to control BRD outbreaks in surplus calves [20]. Selection pressure exerted by antimicrobial therapy was shown to influence the prevalence of resistance among commensal and pathogenic respiratory bacteria. Table 2 represents major respiratory pathogens and associated resistance phenotypes isolated from surplus calves.

*Pasteurella multocida* was most frequently isolated (37%) from veal calves in cases associated with BRD followed by *Mannhemia* spp. [55]. Interestingly, a majority (71.9%) of the resistant strains of the *Pasteurellaceae* family, and isolates with multi-resistance profiles were restricted to organisms originating from veal calves when compared to the dairy and beef production systems [55]. Similar results were obtained in another study where a substantially higher proportion of AMR *P. multocida* and *M. haemolytica* were isolated from intensively reared veal calves in contrast to the more extensively raised beef herds [41]. Antimicrobial susceptibility profiles demonstrated that more than 80% of the resistant *Pasteurellaceae* strains from veal calves were multidrug-resistant (resistant to four or more antimicrobials). A higher percentage of resistant *Pasteurellaceae* strains were recovered from veal calves in Switzerland. The study showed that 85% and 95% of *M. haemolytica* and *P. multocida* were resistant to at least one of the tested antimicrobials [59]. However, Schönecker et al. [58] reported an overall decrease in resistance in *M*. *haemolytica* isolates from veal calves in Switzerland when compared to previous studies [59,62]. Even though there was a decrease in resistance in *M. haemolytica*, no information was available on the health and treatment status of the calves in the study. Among *P. multocida*, reports from Swiss veal calves [41] demonstrated a common occurrence of resistance to tetracyclines and macrolides. More recent studies showed a higher prevalence of resistance among *Pasteurella spp.* To fluoroquinolones and macrolides [58] when compared to earlier studies that had distinctly less resistance towards these antimicrobials [62,63]. Further, group treatment was associated with increased odds of isolating resistant *M. haemolytica* and *P. multocida* isolates [59]. Adding to this, another study in veal calves demonstrated large deviations from recommended dosing regimens, with 88% of oral administrations being underdosed [41].

As for *Mycoplasma bovis* infection, there is no effective vaccine at present, and control is mostly dependent on a variety of husbandry and infection control practices. However, due to the lack of cell wall and its inability to synthesize folic acid, *Mycoplasma* is intrinsically resistant to beta-lactams and sulfonamide classes of antimicrobials. Hence, antimicrobials that target protein or DNA synthesis (such as fluoroquinolones and macrolides) are commonly used for the treatment of *Mycoplasma.* However, resistance against these classes of drugs is frequently reported in veal calves [64]. A significant increasing trend in MIC values of macrolides, that are the first choice for the treatment of BRD caused by *Mycoplasma* spp. Was reported in Netherlands from *M. bovis* collected from clinical samples between the period 2008–2014 [60]. Similarly, a recent study comparing the antimicrobial susceptibility of *Mycoplasma bovis* isolated from dairy, beef, and veal cattle sectors in Belgium showed high percentages (50–100%) of acquired resistance for macrolides. However, no significant differences were observed in AMR between production systems except for gamithromycin which was higher in beef cattle [61].

Overall, a higher percentage of AMR respiratory pathogens were isolated from veal calves when compared to dairy and beef cattle [41,55,56]. Furthermore, there have been drastic changes in the prevalence of resistance among respiratory pathogens among surplus calves. Specifically, a trend of elevated resistance towards the macrolide class of antimicrobials in *Pasteurellaceae* family and *Mycoplasma* spp. Were observed in recent years. Increased resistance towards macrolides in respiratory pathogens does not come as a surprise, since macrolides (such as tilmicosin and tulathromycin) are frequently administered either prophylactically, metaphylactically, or therapeutically in animals recognized to be at high risk for BRD. The high levels of resistance in animal respiratory pathogens are likely to have a substantial but unmeasured impact on calf health and welfare due to treatment failures. Given the fact that surplus calves are an important reservoir of AMR respiratory bacteria, preventive measures against BRD should be revisited, with good management practices implemented at the farm of origin of the surplus calves, long before the arrival at the fattening unit, rather than actions aimed at limiting the spread of the diseases.

### 3.2. Enteric Bacteria in Surplus Calves Contribute to Antimicrobial Resistance Burden

Enteric bacteria, such as *E. coli,* are known to be reservoirs of AMR genes as shown in Table 3. Studies show that surplus calves can carry higher levels of resistant *E. coli* strains compared to older animals [65,66,67,68,69]. For instance, the proportion of extended-spectrum beta-lactamase (ESBL) carrying *E. coli* isolates was higher in veal calves relative to adult cattle in France [32]. Further, Schönecker et al. [59] demonstrated that ~70% of the *E. coli* isolated from veal calves were resistant to at least one of the tested antimicrobials. However, only a few studies studied the prevalence and distribution of AMR in *E. coli* within surplus dairy calves in the U.S. In this context, Salaheen et al. [70] showed that multidrug-resistant *E. coli* (resistant to more than three antimicrobial classes) was isolated from 75–100% of veal calf samples in Pennsylvania. The authors showed that *E. coli* isolated from those calves were resistant to antimicrobials belonging to multiple antimicrobial classes [70]. Hutchinson et al. [71] also documented a higher percentage of multidrug resistance (97%) among *E. coli* recovered from farm isolates in a vertically integrated veal production system in the U.S. Consistent with the study in veal calves in Europe [32,72], resistance to quinolones and macrolides remained low at all times among enteric *E. coli* in the U.S. Similarly, Berge et al. [73] reported lower resistance percentages to quinolones among veal calves in California. Furthermore, a follow-up study from the same authors [3], characterizing the gut microbiota of veal calves by shotgun metagenomic sequencing showed that 70% of veal calves had resistomes containing AMR genes conferring resistance to aminoglycosides, tetracyclines, and MLS.

Studies also show age-related changes in the fecal carriage of AMR in *E. coli* among surplus calf production systems [69]. For instance, resistance to critically important antimicrobials (quinolone resistance) decreased significantly by the end of the fattening process in veal calves in Europe [32]. However, resistance towards non critically important antimicrobials and MDR in *E. coli* increased in parallel to a decrease in resistance to critically important antimicrobials by the end of the fattening process. These results imply an increase in the use of non-critically important antimicrobials during the fattening process. Concurring with these observations, higher tetracycline use was reported in fattening farms when compared to the farms of birth [32]. In contrast, higher quantities of antimicrobial administration were observed in the first three weeks of rearing in veal calves in Ohio [18]. Similar to the studies in Europe, studies in U.S. have found an age-related decline in the carriage of ampicillin-resistant *E. coli* in calves sampled within the first month after birth until up to the eighth month of sampling [74]. In contrast, Salaheen et al. [70] observed higher percentages of resistance in samples collected from animals before slaughter (16–18 weeks) when compared to samples collected from bob calves (<1-month-old) at the auction houses. Further, fluoroquinolone resistance was found in 75% of farm fecal isolates, despite veal calves never receiving fluoroquinolones from veal growers [71]. It is not clear why fluoroquinolone resistance was found in *E. coli* isolates recovered from veal calves. One possibility is that the resistant bacteria were acquired at birth from the dam or dairy-farm environment, as fluoroquinolones are not used in U.S. veal operations. Alternatively, contact with other animals or contaminated environments during auction or transport could have resulted in colonization. Similarly, another study showed the presence of *E. coli* strains with a similar resistance pattern (resistance to ampicillin, streptomycin, sulfisoxazole, tetracycline, and trimethoprim-sulfamethoxazole) isolated from both auction houses and farms suggesting a common source of origin of the isolates [70].

Resistance towards extended-spectrum cephalosporins is a common occurrence in young dairy calves. Despite their public health significance, fecal carriage of extended-spectrum cephalosporins/*Amp*C is not well described in surplus calves. The presence of *bla*_CTX-M_, a group of class A ESBL, conferring resistance to second and third-generation cephalosporins was observed in 13.3–17.5% of veal calf samples in the U.S. [70]. In a similar study from Pennsylvania, Donaldson et al. [75] reported that 100% of *E. coli* isolated from dairy calves that were resistant to ceftiofur were also MDR. Additionally, Hordijk et al. [66] evaluated the fecal samples taken from veal calves and found that 83% of the *E. coli* isolates that were resistant to cefotaxime carried the *bla*_CTX-M_ gene family. Researchers in France also identified CTX-M group 1 enzyme (71.5%) in veal calf isolates with ESBL phenotype. [32]. This is consistent with the data from other European countries where *bla*_CTX-M-1_ was identified as the main gene responsible for ESBL spread in food-producing animals including veal, poultry, cattle, and swine [66,76,77]. Interestingly, 26% of the *E. coli* that harbored ESBL/*Amp*C genes carried multiple plasmid types (both IncI1 and IncF plasmids) within the same isolate, suggesting horizontal gene transfer events occurring within those isolates. Further, the authors also reported a rare combination of *bla*_CTX-M-1_ with IncF-type plasmid in ESBL *E. coli.* [76].

Overall, a higher prevalence of resistance observed among enteric bacteria in young surplus calves relative to older calves is a consistent feature across Europe and the U.S. This can be attributed to several factors such as increased fecal–oral transmission, in vivo fitness advantage of resistant *E. coli* in neonatal calves, or due to the higher levels of AMU in younger animals due to the increased infection risk in those animals [78,79]. Age related changes in the occurrence of AMR is a common feature among calves which is partially a result of changes in management practices and reduced incidence of disease in older calves. Furthermore, the emergence of enteric *E. coli* carrying ESBL such as *bla*_CTX-M-55_ in surplus calves is of significant concern as those isolates are known to carry horizontally transferable genes which mediate resistance to aminoglycoside (*rmtB*) and colistin (*mcr*-3) [80]. High trafficking of calves from different locations across dairy farms along with the presence of resistant determinants capable of horizontal transfer between bacterial populations might have resulted in a diverse set of plasmid/gene combinations among surplus calves. In any case, resistance to CIA antimicrobials among enteric commensal organisms has significant public health implications due to their ability for the widespread dissemination of AMR genes among animal and human bacterial populations.

### 3.3. Major AMR Foodborne Pathogens Recovered from Meat from Surplus Calves

Integrated surveillance efforts have been used to monitor AMR transmission from food animals to humans. This includes sampling on-farm, harvest facilities, and from retail cuts of meat [81]. Much information on AMR bacteria in ground beef and other food products are available. However, information on AMR bacteria in surplus dairy and veal meat is very limited. The first estimates of bacterial contamination and AMR prevalence in retail veal meat in the United States were generated as part of the 2018 U.S. National Antimicrobial Resistance Monitoring System (NARMS) [82]. The study showed that 14% of the *E. coli* and 39% of *Enterococcus* veal-derived isolates were resistant to antimicrobials, and resistance was more likely to be found in isolates derived from veal samples compared to dairy cattle samples [82]. Although a wide variety of AMR bacteria of public health significance are detected in veal meats, major AMR foodborne pathogens of concern include *Salmonella*, *Escherichia coli*, and *Campylobacter* [83].

MDR (resistant to five or more antimicrobials) *Salmonella* was detected in 24% of the total *Salmonella* isolated from grain-fed veal meat [84]. Studies also show that bob veal samples harbored higher concentrations of *Salmonella* compared to special-fed veal [85]. While *Salmonella* isolated from healthy adult cattle are typically pan susceptible and are not of high public health significance [86], the opposite is true for *Salmonella* isolated from surplus calves. For instance, special-fed veal was found to harbor *Salmonella* serovars of greater clinical importance (such as monophasic Typhimurium 4,[5],12:i:-, Heidelberg, and Agona) than those recovered from bob veal samples. Additionally, *Salmonella* recovered from special-fed veal was found to be more resistant than those from bob veal [85]. Further, samples from mesenteric lymph nodes collected from a vertically integrated veal production company revealed the presence of *Salmonella* in 21.9% (35/160) of the samples [87]. In addition, serotypes of high public health importance were identified in the study and include *Salmonella* Typhimurium, and Newport [87]. MDR *Salmonella* Dublin was also recovered from ground veal [82]. *Salmonella* Dublin is a cattle-adapted serovar with the potential for causing severe disease in humans [88]. The incidence of human Dublin infections has been increasing over the last few decades [89] and multidrug resistance is a hallmark of American isolates [90]. Work within a veal production company revealed that over 70.8% of lymph node isolates (34/48) were MDR and *Salmonella* Dublin was the most common serovar recovered from the samples. [91]. Further genomic analysis of Dublin isolates recovered from those veal lymph nodes suggested lymph node infection that could be traced to the farm of origin [91].

Similar to the occurrence of resistance in *Salmonella,* AMR was also reported in *E. coli* and *Campylobacter* in veal meat. Resistance to five or more antimicrobials was detected in 33% and 10% of the total *E. coli* isolates isolated from milk-fed and grain-fed veal samples, respectively [84,92]. Work within a veal production system in the U.S. that harvested bob and special-fed veal recovered MDR (resistance to three or more antimicrobial classes) *E. coli* from 61% (51/84) pre-evisceration isolates, and 22% (5/21) final carcass isolates [71]. Additionally, an epidemiologic investigation to identify the source of sporadic Campylobacteriosis outbreaks in humans linked the veal liver as a potential source of infection [93]. In a study in Switzerland, 27% of the *Campylobacter spp*. isolated from veal calves at slaughter were resistant to at least one of the tested antimicrobials [94]. A low prevalence of *Campylobacter* was observed in retail grain-fed veal meat in Canada. However, 50% of the samples were resistant to one or more tested antimicrobials [94].

To summarize, antimicrobial resistance is typically found in isolates recovered from surplus calves. The consistent recovery of MDR *Salmonella* and *E. coli* from veal meat implicates this population as a reservoir for MDR pathogens. Furthermore, the *Salmonella* serotypes that are recovered from surplus calf meat have higher public health importance relative to isolates from other cattle production classes. Surplus veal calves (both bob and formula-fed) are unique populations with differing challenges from beef cattle, but the lack of knowledge on the source and prevalence of AMR foodborne pathogens in surplus calf meat hinders the creation of appropriate interventions. Therefore, to assess potential food safety risks and efficiently target mitigation efforts, further research into surplus calf meat production is prudent.

## 4. Antimicrobial Resistance Control Strategies Focused on Reduced Antimicrobial Use in Surplus Calf Production

Given the high levels of AMR within bacteria from surplus calves and the likely, albeit unmeasured public health impact, improvements in antimicrobial stewardship among veal and dairy beef farms is necessary for long-term sustainability and maintenance of a social license to operate [4,95]. The most often stated goal for antimicrobial stewardship programs is to reduce the total quantity of antimicrobials used throughout the production process [96]. Based on limited evidence in North America, veal and dairy-beef production systems have higher levels of AMU dosing rates relative to other cattle production systems, and a higher prevalence of AMR commensals and pathogens relative to other production classes of cattle. Undoubtedly, reducing AMU and/or AMR will be particularly challenging within surplus calf production systems. Reductions in the total quantity of AMU can be achieved through some combination of reduced disease incidence and judicious (i.e., selective) antimicrobial use [23,31,97,98]. However, the disaggregated nature of the production chain makes changes difficult. For instance, dairy farmers responsible for early-life care have little financial motivation for investments in the care of calves sold at auctions [4,9]. Collaboration across sectors of the production system will be necessary to remove some of the causes of disease. Conflicting messages and a lack of harmony among different advisors were major barriers to applying novel management practices to reduce unnecessary use [99,100]. Additionally, high production costs combined with unstable calf prices, calf quality, and scarce labor keep surplus calf producers under constant financial pressure [101], which makes motivating changes difficult.

Improving Judicious Application of Antimicrobials

**Figure 2:**
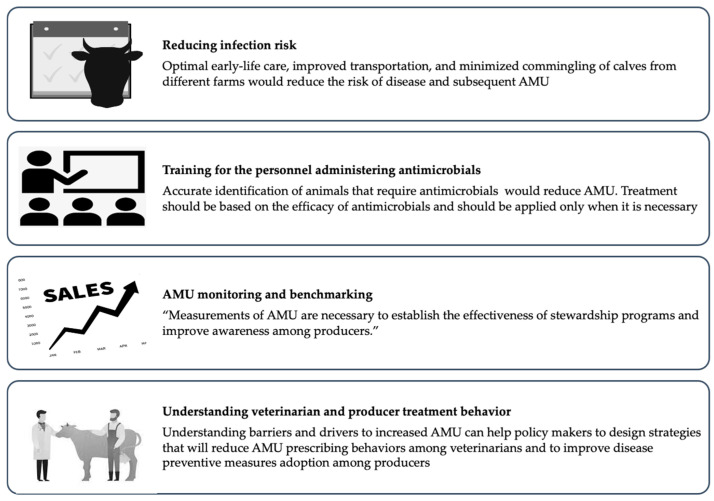
Strategies for reducing antimicrobial use in surplus calf production.

In addition to reduced disease incidence, reductions in AMR can be achieved by adopting strategies for judicious AMU (Figure 2).

Selective application of antimicrobials or reserving AMU where it is necessary for animal health is likely to be effective in reducing AMU. However, some routine applications of antimicrobials lack documented evidence of efficacy. Metaphylactic treatment based on oral administration of antimicrobials for healthy and diseased animals within the same group can be a frequent practice [42], and for instance, U.S. producers sometimes use extra-label administration of antimicrobials in the water to treat or prevent gastrointestinal disease. The efficacy of this practice has not been documented, and optimal antimicrobial stewardship will require evidence to support routine use. Similarly, appropriate training is necessary for farm personnel to accurately identify instances requiring antimicrobial therapy and to follow veterinary written treatment protocols. Two-thirds of veal producers in the Midwestern United States reported routinely using antimicrobials when presented with uncomplicated cases of diarrhea that could be managed with non-antimicrobial interventions [102]. Indeed, training on the accurate identification of calves with diarrhea and pneumonia led to better-targeted therapy and a 50% reduction in the quantity of antimicrobials on Ohio veal herds [30]. Routine implementation of more selective application of antimicrobials will require an additional understanding of behavioral drivers and barriers among farm personnel and veterinarians responsible for AMU. Research has begun to identify the drivers and barriers that influence veterinarians’ and farm owners’ intentions to make treatment decisions [103]. The risk aversion to the high mortality rate among veal calves has been found to facilitate the start of antimicrobial therapy among veterinarians in Flanders, Belgium [20]. Additionally, veterinarians acknowledged that they were afraid of legal action from their employers in the event of negative clinical outcomes if they refuse to treat with antimicrobials [103]. Veterinarians wanted to make responsible usage of antimicrobials, but sometimes they responded to the uncertainty of clinical cases and the need for favorable results by prescribing antimicrobials [104].

Furthermore, routine measurement and monitoring of antimicrobial consumption are necessary to document patterns and temporal changes in the consumption of antimicrobials. Standardized and consistent methods for AMU monitoring have not yet been adopted in the U.S, but the methods will need to be scalable, efficient, and able to document farm-level variability. Importantly, systematic monitoring should protect the confidentiality of participant farms. Candidate methods for AMU monitoring on cattle operations in the U.S. are either based on farm treatment records [105] or sales data [106]. Using treatment records to quantify AMU would be particularly challenging on surplus calf operations as meat residue violations are not a concern for early life treatments, and treatments are recorded less frequently relative to other cattle production classes. Therefore, veterinary sales data are likely the only viable option, which would be enabled by the forthcoming FDA rules eliminating over-the-counter sales of medically important antimicrobials [107]. Given the availability of consistent farm-level AMU measurements, benchmarking tools could be used to improve the awareness of producers on the quantity of AMU and motivate the behavioral changes necessary for meaningful reductions [108,109].

A combination of contextual and psychosocial factors serves as important barriers to a reduction in AMU either through reducing disease incidence or improving selectivity for application of antimicrobials. Future research incorporating social science approaches is necessary to understand the barriers to the implementation of antimicrobial stewardship. Coordination across components of the surplus calf production chain and collaboration between actors in the industry, academics, and regulators are additionally necessary to facilitate or implement stewardship activities, including antimicrobial use monitoring.

## 5. Conclusions

Surplus dairy calves are likely an underrecognized source of AMR bacteria, and reducing the global AMU in the surplus calf sector will be important to mitigate public health risks and assuage consumer concerns. Several challenges faced by surplus calves during their early life (such as long-distance transportation, commingling, and inadequate nutrition) predispose them to respiratory and enteric diseases, necessitating relatively high dosing rates of antimicrobials. Treatment strategies in veal calves often depend on group administration of antimicrobials rather than treating calves individually. Very few studies have characterized AMU in North American surplus calf production systems, and the lack of standardized dosing metrics for AMU quantification hinders AMU reduction strategies. A variety of factors, including young age, high exposures, and antimicrobial use result in substantial levels of resistance to medically important antimicrobials. Coordination across the disaggregated sectors of surplus calf production will be required to reduce disease incidence and AMU. A better understanding of psychosocial and contextual barriers faced by veterinarians and farm owners will facilitate the implementation of economically feasible antimicrobial stewardship practices that maintain animal health and welfare. However, it is still unclear to what magnitude surplus calves affect AMR in livestock production; therefore, additional work will be necessary to characterize and mitigate the impact on public health.

## Figures and Tables

**Table 1:** Risk factors contributing to increased antimicrobial use in surplus calves.

	Factors that Increase Antimicrobial Use Risk	Reference
Antimicrobial Use	1. Winter months	[20]
	2. Beef breed surplus calves compared to dairy and crossbred	[20]
	3. Higher the number of source farms	[42]
	4. Higher ammonia levels	[42]
	5. Practices such as health checks and quarantine reduce the risk of AMU	[31]
	6. A higher number of calves/drinking nipple	[42]
	7. Lower body weight	[12,25,43]
	8. Younger age at arrival	[24,44,45]
	9. Calf health—Diarrhea, cough, pyrexia, depressed attitude, umbilical infection, dehydration, and failed transfer of passive immunity	[9,25,46,47,48]

**Table 2:** Summary of research that studied resistance among bovine respiratory pathogens in surplus calves: The table below shows the source of the different isolates included in the study, country in which the study was conducted, panel of antimicrobials that were tested against the bacterial agent and, major findings from the study.

Bacterial Agents Studied	Country	Antimicrobials Tested	Major Findings	Reference
*Pasteurellaceae* (*P.multocida*, *Pasteurella* spp, *M*. *haemolytica*, *Mannheimia* spp.) from the respiratory tract of healthy veal calves	Belgium	Ampicillin, ceftiofur, oxytetracycline, gentamicin, enrofloxacin, tilmicosin, trimethoprim-sulfadimidine	Acquired resistance to ampicillin, oxytetracycline, trimethoprim-sulfadimidine, gentamicin, tilmicosin, enrofloxacin more common in veal herds when compared to dairy and beef	[55]
*P. multocida*, *M. haemolytica* from respiratory samples collected during two time points (at 4 and 24 weeks after arrival at a veal farm)	Belgium	Ampicillin, amoxicillin-clavulanic acid, ceftiofur, oxytetracycline, trimethoprim-sulfonamide, neomycin, gentamicin, spectinomycin, nalidixic acid, flumequine, enrofloxacin	Veal herds had a substantially higher number of resistant isolates compared to beef herds. Antimicrobials to which highest percentages of resistance identified include -ampicillin (8–30%), tetracyclines (38–43%), trimethoprim sulfonamide (30–50%), nalidixic acid (19–49%), flumequine (16–44%), enrofloxacin (10–36%), neomycin (18–45%), streptomycin (78–89%), gentamicin (15–43%)	[41]
*M. haemolytica,* *P. multocida* from the respiratory tract of veal calves	Switzerland	Ceftiofur, danofloxacin, enrofloxacin, tilmicosin, tulathromycin, spectinomycin, penicillin, oxytetracycline, florfenicol, ceftiofur,	Antimicrobials to which highest percentages of resistance identified include—oxytetracyclines (27–94%), penicillin (42–52%), spectinomycin (0.3–81%), tilmicosin (53%), tulathromycin (0–30%) and danofloxacin (14–36%)	[59]
*P. multocida,* *M. haemolytica,* *Histophilus somni* * from the nasopharynx of young and older veal calves	Switzerland	Ceftiofur, danofloxacin, enrofloxacin, tulathromycin, spectinomycin, penicillin, oxytetracycline, florfenicol, tilmicosin	AMR was common against oxytetracycline, spectinomycin, tulathromycin, penicillin and danofloxacin	[58]
*Mycoplasma bovis* from clinical samples	Netherland	Enrofloxacin, erythromycin, oxytetracycline, tilmicosin, tulathromycin, tylosin, ampicillin, ceftiofur, chlortetracycline, clindamycin, danofloxacin, florfenicol, gentamicin, neomycin, penicillin, spectinomycin, sulphadimethoxine, tiamulin, trimethoprim-sulphamethoxazole	The highest minimum inhibitory concentrations (MIC) values were obtained for erythromycin, tilmicosin, tylosin.	[60]
*M. bovis* from respiratory samples	Belgium	Florfenicol, oxytetracycline, doxycycline, tilmicosin, tylosin, gamithromycin, tiamulin, gentamicin, enrofloxacin	No significant difference in resistance was observed between veal, dairy and beef herds except for gamithromyicn (highest resistance in beef herds). Higher MIC values were obtained for tilmicosin, tylosin, gamithromycin and florfenicol	[61]

* No resistance was detected in *H. somni.*

**Table 3:** Summary of research that studied resistance among enteric bacteria in surplus calves: The table below shows the source of the different isolates included in the study, the country in which the study was conducted, the panel of antimicrobials that were tested against the bacterial agent, antimicrobials to which highest proportion of resistance was observed and major antibiotic resistance genes that were identified in the study.

Isolates Studied	Country	Antimicrobials Tested	Antimicrobials to Which the Highest Proportion of Resistance Was Observed	Major Antibiotic Resistance Genes Identified	Reference
ESBL/*Amp*C producing *E. coli* from fecal samples collected from veal calves upon arrival at the fattening farm and just before departure to the slaughterhouse	France	Amoxicillin, amoxicillin-clavulanic acid, cefalothin, cefuroxime, ceftiofur, cefoxitin, cefquinome, ertapenem), tetracycline, gentamicin, streptomycin, florfenicol, colistin, sulfonamides, nalidixic acid, enrofloxacin	Amoxicillin (69%)-, tetracyclines (90–93%), streptomycin (74–80%), sulfonamides (78–95%)	CTX-M group 1 (*bla*_CTX-M-1_, *bla*_CTX-M-15_, *bla*_CTX-M-32_, *bla*_CTX-M-55_, *bla*_CTX-M-3_) group 9, group 2, *bla*_CMY-2_, *mcr-1*, *mcr-3*	[32]
*E. coli* from calves from multiple veal farms	Switzerland	Ceftiofur, enrofloxacin, gentamicin, neomycin, spectinomycin, ampicillin, oxytetracycline	Oxytetracycline (66%), ampicillin (54%), neomycin (26%), spectinomycin (25%), gentamicin (15%), enrofloxacin (14%)	Not studied	[59]
ESBL*/Amp*C *E. coli* from veal calf fecal samples collected from 1997 to 2010	Netherland	Tested only for cefotaxime susceptibility	-	*Amp*C type 3, type 34, *bla*_CMY-2_, *bla*_CTX-M-1_, *bla*_CTX-M-2 or 97_, *bla*_CTX-M-14_, *bla*_CTX-M-15_, *bla*_CTX-M-32_, *bla*_CTX-M-79_, *bla*_TEM-52_, *bla*_TEM-20_, *bla*_SHV-12_	[66]
*E. coli* from manure and fecal samples from calves in auction houses and at veal calf operations	U.S.	Ampicillin, amoxicillin-clavulanic acid, cefoxitin, ceftiofur, ceftriaxone, gentamicin, sulfisoxazole, trimethoprim-sulfamethoxazole, azithromycin, chloramphenicol, tetracycline, streptomycin, ciprofloxacin, nalidixic acid	Tetracyclines (75–100%), Penicillins (50–100%), Aminoglycosides (60–100%), Phenicols (25–100%), Folate pathway inhibitors (40–100%), Beta-lactams (20–100%)	*bla*_CTX-M-1_, *bla*_CTX-M-9_	[70]
Fecal microbial community of commercially raised veal calves early and late stages of production (metagenomic study)	U.S.	Not tested	-	* ARGs to aminoglycosides, tetracyclines, macrolide-lincosamide-streptogramin B	[3]
*E. coli* from feces and carcass swabs from a vertically integrated veal production system	U.S.	Ampicillin, ciprofloxacin, ceftriaxone, chloramphenicol, cefoxitin, gentamicin, neomycin, nalidixic acid, streptomycin, sulfamethoxazole-trimethoprim, tetracycline, ceftiofur	Ampicillin (25–95%), neomycin (20–98%), streptomycin (30–95%), tetracycline (45–98%), sulfamethoxazole-trimethoprim (20–92%)	*bla*_CMY-2_, *bla*_CTX-M_ (only two genes were tested)	[71]

* Too many ARGs were present to list out separately.

## Data Availability

Not applicable.
